# Epidemiology of traumatic injuries and associated infectious complications in the Republic of Kazakhstan

**DOI:** 10.25122/jml-2021-0377

**Published:** 2022-04

**Authors:** Natalya Pliska

**Affiliations:** 1. Immunobacteriological Laboratory, National Scientific Center of Traumatology and Orthopedics, Nur-Sultan, Republic of Kazakhstan

**Keywords:** injury epidemiology, dynamics, incidence, osteomyelitis, surgery

## Abstract

Traumatism is one of the most important contemporary medical and social issues for most countries worldwide. Since the 20^th^ century, the urgency of traumatism has been increasing. There was an increase in fatal traumatism, including non-fatal cases resulting in permanent disability or temporary disability. This study aimed to investigate the epidemiology of injuries in the Republic of Kazakhstan and identify the statistical patterns of surgical treatment. Furthermore, this study aimed to identify the incidence of infectious complications in patients who received trauma and orthopedic care, their structure and dynamics, and compare this data with the literature. From 2017 to 2019, there were more upper and lower extremity injuries in the Republic of Kazakhstan in the age group of 15–17 years, which corresponds to Russian statistics. Of the 10 injuries, one in three undergoes surgical intervention. In two large cities, Nur-Sultan and Almaty, surgical interventions are performed more often than in other regions. The most frequent infectious complication associated with traumatism is osteomyelitis, with the most causative species being staphylococci.

## Introduction

The concept of traumatism includes a set of injuries that occurred in a certain group of the population over a certain period. Trauma is covered in the study of epidemiology, which assumes a conditioned interdependence, studies the causality between this incident (injury) and the external environment or internal state of the victim's body. The causality is identified by constant monitoring and studying the conditions and circumstances of the injury occurrence, with further analysis of all the causes and factors that determine their regularity. Traumatism is one of the most important contemporary medical and social problems in most countries [1]. Since the 20^th^ century, the urgency of traumatism has been increasing. An increase in fatal traumatism was noted, including non-fatal cases resulting in permanent disability or temporary disability.

**Figure 1.**
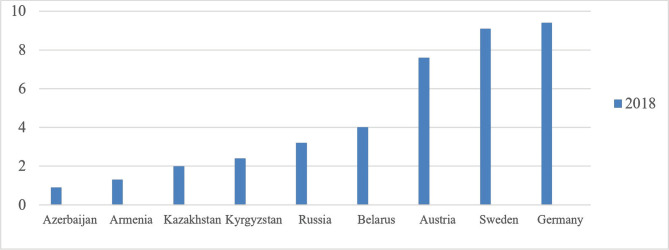
Funds distribution for the development of medicine in terms of GDP.

The development of traumatology, as well as medicine as a whole, largely depends on budgetary funds allocated by the state in terms of gross domestic product (GDP) per capita in various states at purchasing power parity (PPP), which is a feature that determines the level of economic development in a certain period. Thus, in 2018, various countries allocated funds for the development of medicine (Figure 1). Over the past three years, the staffing level in the Republic of Kazakhstan remained at the level of 0.6 per 10 thousand population. Almost all regions are below the level of the republican indicator, except for four large cities: Nur-Sultan, Almaty, Pavlodar, and Karaganda. Naturally, the hospital supply for highly specialized trauma and orthopedic care is concentrated in these cities, accounting for 29.2% of 17 regions. The number of surgical operations performed every year on average increases by 2.8% [1].

Various complications may arise following surgical and orthopedic care. This study investigated one of the later etiologically monitored infectious inflammations – osteomyelitis. Complications in the treatment of injuries have been known since the end of the 19^th^ century. Thus, in 1880, Louis Pasteur isolated a microbe from the purulence of a patient with osteomyelitis and named it *staphylococcus*. Later it was found that any pyogenic microbes can cause osteomyelitis [2]. At present, musculoskeletal system infections are widespread, accounting for up to 10% of the overall structure of morbidity. Moreover, there is no tendency to decrease despite doctors' new technologies and treatment methods in many countries – the costs of treatment, rehabilitation, and permanent disability of patients are increasing [3]. 

Today, according to the WHO, over 2,000 people die every day due to injuries, 60 thousand people go to hospitals, and 600 thousand are forced to seek emergency outpatient care in economically developed countries. Injuries rank third among the causes of death, mainly among working-age people. The highest level of traumatism is observed in men aged 20–49 years and in women aged 30–59. Furthermore, this indicator is much higher in men in all age groups [4]. The traumatism rate in the Commonwealth of Independent States (CIS) is one of the highest in the world and continues to deteriorate, while in the European Union (EU), it is one of the best in the world and continues to improve. On average, the risk of dying from traumatism for men in the CIS is almost 4 times higher than men in the EU. At the initiative of the Eurobureau, the WHO redistributed the treatment costs of the population, shifting them to the wealthier segments of society to reduce the burden of costs for the poor [5]. The epidemiological situation of injuries in Russia is extremely tense. More than 12 million cases of injuries and poisoning are registered in the country annually, of which 93% are injuries, 1% are poisoning, and 6% are other accidents. The average level of injury is 120–130 cases per 1,000 population [6, 7].

The purpose of the paper was to investigate the epidemiology of injuries in the Republic of Kazakhstan and identify the statistical patterns of surgical treatment. Furthermore, this study aimed to identify the incidence of infectious complications in patients who received trauma and orthopedic care, their structure and dynamics, and compare this data with the literature.

## Material and Methods

The epidemiological analysis of injury indicators in Kazakhstan is compared with data from Russia from the last three years. The selection of biological material from foci of purulent infection in patients with osteomyelitis was carried out on a follow-up basis. The material was taken from patients upon admission (on the first day of hospitalization), next – in the dressing room, during dressings, or during other medical and diagnostic procedures. Data included official statistics on diseases associated with traumatism and the occurrence of infectious complications among the adult population of the Republic of Kazakhstan for 2017–2019 [8]. In addition, a retrospective analysis of the microbiological examination of biomaterial from patients and the isolation of the main causative agents of infectious inflammation was carried out. The object of the study was smeared from the wound fluid, fistulous canal, puncture sample, and the biomaterial sampled after surgery in the case of repeated revision of the joint or its replacement. Patients from all regions of Kazakhstan admitted with chronic osteomyelitis were included in the study from the Republican State Enterprise Research Institute of Traumatology and Orthopaedics (NIITO) in Nur-Sultan between 2017–2019.

The biomaterial was examined by conventional methods, and it was mandatorily subjected to quantitative bacteriological research. The primary inoculation of the biomaterial was performed on various nutrient media, staining of smears was carried out according to Gram, the species identification of the isolated microorganisms was performed by the classical bacteriological method for the study of morphological, cultural, and biochemical properties [8].

## Results

**Figure 2.**
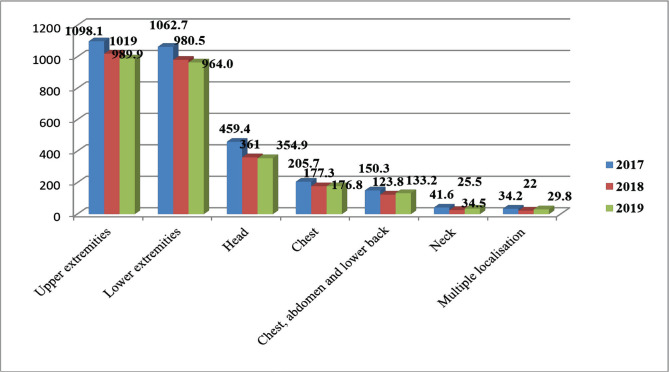
Intensity indicator of traumatism of various localizations between 2017 and 2019 in the Republic of Kazakhstan.

According to official statistics, in total, over the past three years in Russia, 24,325.2 cases of injuries per 100 thousand of the population were registered, *i.e.*, the intensity indicator for the average number of injuries received per year amounted to 8,108.4 [8]. From 2017 to 2019, 1,542,374 cases of mechanical injuries in various localizations were registered in the Republic of Kazakhstan in absolute numbers. On average, half a million cases of injuries of various localizations are registered in Kazakhstan (514,124 cases – the average number for three years) every year, registered in all age groups. The intensity indicator for the analyzed period is, in total, 8,444.2. The average for this period is 2,814.7. In 2019, it ranged from 3,052 to 2,683 (Figure 2). Regarding the dynamics of injury within the Republic of Kazakhstan for three years, there was a change in the intensity indicator, which is calculated per 100 thousand of the republic's population. There are pronounced downward trends with an annual rate of 4.02% per year. The incidence rate indicator in 2019 decreased by 12.1% compared to 2017, and in comparison with the data from the Russian Federal State Statistics Service, injuries in Kazakhstan in terms of the total number are 2.88 times lower.

**Figure 3.**
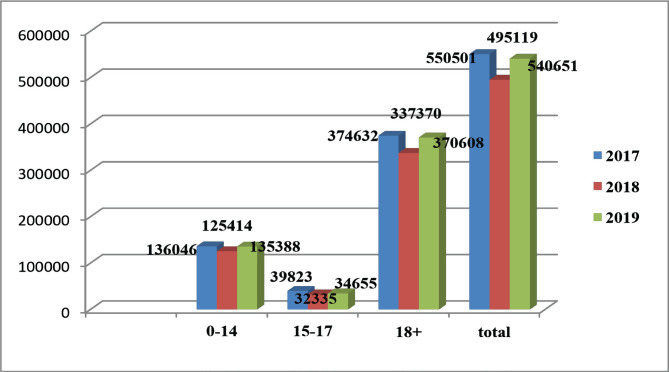
The incidence of traumatism in 2017–2019 by age in absolute numbers and in total.

According to statistics in Russia (2016–2018), there were officially registered: 25,234 patients with injuries, poisoning, and some other consequences of external causes in 2016, 25,127.4 in 2017, and 25,346 in 2018 per 100 thousand people. When comparing the data on injuries in Russia over the past three years, injuries of the upper extremities are also leading in this country. Moreover, this localization is 2.6 times higher than in Kazakhstan. In the second place, as in Kazakhstan, are injuries of the lower extremities, but according to the number of injuries, they differ from injuries of the upper extremities – only by 4%. In comparison with Kazakhstan, this indicator is 2.6 times higher in Russia. Head injuries are in third place, but this figure is 2.6 times higher in Russia. Figure 3 presents the age groups of the population in the Republic of Kazakhstan who were injured under the influence of various physical factors.

Compared with Russian statistics, in children aged 0 to 14 years, the incidence does not change during the observed time, with wave-like fluctuations of 3.5%. Analysis of the incidence of traumatism in children with various causes by year indicates that in 2016 there were 10,403.5 cases, in 2017 – 10,251.7 cases, in 2018 – 10,618.3 cases, respectively, per 100 thousand children. Another group is adolescents aged 15–17 years, where the incidence of traumatism per year is as follows: 2016 – 17,473.7, 2017 – 17,393.3, 2018 – 17,438.6. A wave-like decrease in the indicator by 0.5% is recorded, which in this case is considered a stable value. In Russia and European countries, the incidence of traumatism in adolescents and young people is higher than in all other age groups [9, 10].

**Figure 4.**
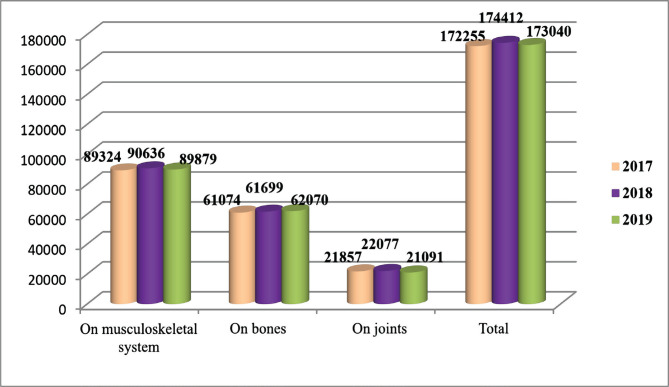
The number of surgical operations performed (for adults and children) between 2017–2018.

In Kazakhstan, only 6–10% of patients injured and seeking medical help require hospitalization with inpatient care. The percentage of all injuries that require surgical treatment (trauma and orthopedic) and the most frequent surgical operations performed during the study period are presented in Figure 4. It is possible to determine the frequency of traumatism cases requiring surgical intervention.

The surgical operations performed during 2017–2018 indicate that most were performed on the musculoskeletal system. The same number of such operations was performed in 2017–2018, which amounts to 51.9% of the total number of operations. In 2017, 35.5% of operations were performed on bones, and in 2018, practically the same number of operations was performed – 35.4%, *i.e.*, 0.1% lower than the previous year. In 2017, joint surgeries were performed with a percentage ratio of 12.6%, and in 2018 – 12.7%. This type of surgical treatment is considered the most time-consuming, costly and requires high professionalism.

Russian patients with injuries required inpatient surgical care on an average of 6.85% of cases (with cases of traumatism in 2016 – 6.9%, in 2017 – 6.8%, in 2018 – 6.9%). The number of operations performed on the musculoskeletal system was more than on other anatomical fragments, with a constant increase of 1.8% per year on average (in 2016 – 1,241.9, in 2017 – 1,271.6, in 2018 – 1313.1). Patients in Kazakhstan and Russia share almost the same proportions between the injuries received and the need for hospitalization. In this case, the anatomical fragments are in the same sequence according to the number of surgical operations performed. Most of these operations were performed on the musculoskeletal system, but the number of operations performed in Russia increased by 1.8%, while in Kazakhstan, it decreased by 0.8%

The highest rates of surgical intervention in all 17 regions of the Republic of Kazakhstan are in the following areas: North Kazakhstan, West Kazakhstan, and Karaganda. In these areas, after three years of monitoring, the highest rates of surgical intervention in cases of traumatism are observed: rates range from 102% to 110.9%. On the contrary, the lowest rates of surgical intervention in traumatology and orthopedics are in the following regions: Turkestan, Atyrau, Almaty, Mangystau, and are registered in the following range: 35.6–56.8%. At the NIITO institute from Nur-Sultan, the rate of surgical intervention is slightly higher than the average of 84.8–85.5% since the institute specializes in highly technological medical manipulations – this is mainly prosthetic care for joints of various localization.

**Figure 5.**
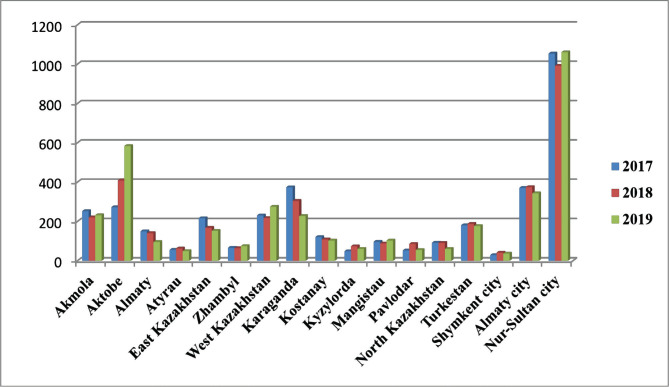
Osteomyelitis treated in various regions of Kazakhstan between 2017–2019.

The total incidence of osteomyelitis registered over the past three years, in absolute terms, amounted to 10,997 cases: in 2017 – 3,667 cases, in 2018 – 3,635 cases, in 2019 – 3,695 cases, respectively, *i.e.*, the amount remains almost unchanged with minor fluctuations of 0.8–1.6%. Figure 5 demonstrates all registered cases by regions of Kazakhstan.

According to Figure 5, the largest number of cases treated of osteomyelitis (992–1,061) can be observed in Nur-Sultan. It can be assumed that since Nur-Sultan is the capital, the specialists there are more erudite, and the reputation of medical institutions is superior. The capital of Kazakhstan is home to the Scientific Research Institute of Traumatology and Orthopedics (NIITO), which has qualitative medical equipment. Since patients in Kazakhstan have the right to choose a medical institution for planned hospitalization, complex cases are more often treated precisely in this institution. Furthermore, NIITO has 14 departments with a narrow focus of each department on the treatment and prosthetic care of a particular anatomical area. The average number of osteomyelitis cases treated in Kazakhstan was registered in Aktobe, Akmola, Karaganda, West Kazakhstan, and Almaty in 2017–2019, amounting to 218–374 cases. In other regions of Kazakhstan, the number of treated patients with this pathology in medical institutions ranges from several dozen cases (49) to less than 200 cases per year in total. During the three years of the study, the incidence of *Pseudomonas aeruginosa* in the wound fluid of patients suffering from osteomyelitis ranks third, where the average value for this period amounted to 10.8% of cultures. The distribution of this pathogen in the wound fluid of patients by year is as follows: in 2017 – 9.4%, in 2018 – 12.5%, in 2019 – 10.5%.

After providing trauma and orthopedic care to patients, the etiology of osteomyelitis infections was as follows: in 2017, 966 studies of biomaterial with this nosological form were carried out, of which 13 were negative, which amounts to 1.3%. In 2018, 967 studies were carried out, 310 (32%) negative results were detected, and in 2019, 1,202 biomaterials were examined, and negative results amounted to 426 cases (35.4%). Over the three years under study, 23 types of microorganisms were isolated in the wound fluid. However, 4 main species played a leading role in the occurrence of osteomyelitis: *Staphylococcus aureus*, *Staphylococcus epidermidis*, *Pseudomonas aeruginosa*, *E. Coli*, which is presented in Table 1.

**Table 1.** The main causative agents of osteomyelitis between 2017–2019.

**Name of microorganisms**	**2017**		**2018**		**2019**		**Total**	
	**abs.**	**%**	**abs.**	**%**	**abs.**	**%**	**abs.**	**%**
**Staphylococcus aureus**	426	44.7	332	50.5	310	42.5	1,068	45.9
**Staphylococcus spp.**	286	30.01	129	19.6	124	17.0	539	23.2
**Pseudomonas aeruginosa**	90	9.4	82	12.5	77	10.5	249	10.7
**Escherichia coli**	42	4.4	37	5.6	35	4.8	114	4.9
**Other microorganisms**	109	11.49	77	11.8	170	23.3	356	15.3
**Total**	953	–	657	–	730	–	2.326	–

Based on the data obtained, it can be concluded that the leading causative agent of osteomyelitis is *Staphylococcus aureus,* with an average total excretion of 45.9%, *i.e.*, half of all isolated microorganisms. Considering the total amount of isolated *staphylococcus* during the study period, the total average amount is 69.1%, *i.e.*, 7/10. *Pseudomonas aeruginosa* was secreted 7 times less than all staphylococci, and *Escherichia coli* – 14 times less. Other microorganisms were isolated in 15.3%, represented by 17 species, which suggests that they were rarely isolated.

## Discussion

The provision of any medical care is evaluated according to the quality of the services rendered. Quality is evaluated according to the recovery and return of organ functions, according to the presence of complications and fatalities. The success of the operation does not always promise a complete recovery, and not everything depends on the professionalism of the doctor, or their competence in treating a certain disease. Some reasons impede recovery and are independent of the medical institution [11]:

• Open wound management;
• Poor quality of implants and medical devices (suture material);
• Duration of hospitalization for more than 50 days;
• More than one surgical intervention in the course of the treatment period;
• Associated diseases (diabetes, complicated hypertension, coronary artery disease, tissue denervation etc);
• The presence of an external fixation device or drainage in the wound;
• The age of patients over 50 years old;
• Gender (men are more prone to complications).

Furthermore, the prerequisites contributing to the occurrence of healthcare-associated infections include insufficient disinfection of hands by medical personnel, lack of personal protective equipment in the course of dressing, dressing outside the dressing room, re-hospitalized patients are brought into the department with strains, intraspecific characteristics formed as a result of preceding medical care. As a result, complications arise in trauma and orthopedics, among which most often are osteomyelitis and periprosthetic infections that occur distantly, *i.e.*, 6–12 months and later [12].

This study recorded two times lower rates of chronic osteomyelitis in adults than the data of Russian authors [13]. There was a change in the intensity indicator, which is calculated per 100 thousand of the population of the republic, a pronounced trend towards a decrease in figures with an annual rate of decrease of 3.4% registered upon monitoring various anatomical areas. In the most vulnerable in terms of injuries and, at the same time, the smallest group of adolescents (aged 15 to 17), the maximum number of registered cases of traumatism was identified for each year of age. Based on the figures obtained, the number of detected cases over the three years fluctuates within small limits, but with a steady decline from 7.3% to 6.5% annually. Based on official statistics for injuries in the Republic of Kazakhstan during the studied period, it can be assumed that all injuries received in 30% (31.3–34.8%) of cases are threatening. These patients need treatment correction in a hospital setting and often undergo surgical treatment. In the structure of traumatology and orthopedics microbial pathogens, the leading positions are occupied by *Staphylococcus aureus* and coagulase-negative staphylococci (CNS), which are represented mainly by *Staphylococcus epidermidis* [11, 12, 14], which is consistent with data from this study.

## Conclusions

The study suggests that the upper and lower extremities are more prone to injuries than other anatomical areas in the Republic of Kazakhstan. Furthermore, injuries are more prevalent among the 15–17 years age group, which corresponds with injury statistics in Russia. Of the 10 injuries, one in three undergoes surgical intervention. Surgical interventions are predominantly performed in two large cities – Nur-Sultan and Almaty. The most common complication is osteomyelitis, in which the most causative type is *Staphylococcus aureus*.

## Acknowledgements

### Conflict of interest

The author declares no conflict of interest.

### Ethics approval

The study was approved by the National Ethics Commission of the Ministry of Health of the Republic of Kazakhstan (No. 169-A, Date: October 14, 2019).

### Authorship

NP contributed to conceptualization, project administration, data curation, formal analysis, methodology, writing, review, and editing.
